# Hydrophobin-Protein A Fusion Protein Produced in Plants Efficiently Purified an Anti-West Nile Virus Monoclonal Antibody from Plant Extracts via Aqueous Two-Phase Separation

**DOI:** 10.3390/ijms21062140

**Published:** 2020-03-20

**Authors:** Collin Jugler, Jussi Joensuu, Qiang Chen

**Affiliations:** 1 The Biodesign Institute and School of Life Sciences, Arizona State University, Mail Zone 5401, 1001 S. McAllister Avenue, Tempe, AZ 85287, USA; 2 VTT Technical Research Centre of Finland Ltd, Espoo, Finland

**Keywords:** monoclonal antibody (mAb), aqueous two-phase separation (ATPS), hydrophobin, West Nile virus, plant-made antibody

## Abstract

The development of monoclonal antibodies (mAbs) has provided vast opportunities to treat a wide range of diseases from cancer to viral infections. While plant-based production of mAbs has effectively lowered the upstream cost of mAb production compared to mammalian cell cultures, further optimization of downstream processing, especially in extending the longevity of Protein A resin by an effective bulk separation step, will further reduce the overall prohibitive cost of mAb production. In this study, we explored the feasibility of using aqueous two-phase separation (ATPS) in capturing and separating plant-made mAbs from host proteins. Our results demonstrated that an anti-West Nile virus mAb (E16) was efficiently separated from most plant host proteins by a single ATPS step, comprising the mixing of plant extracts containing Hydrophobin-Protein A fusion protein (HPA) and E16 and the subsequent incubation with an inexpensive detergent. This simple ATPS step yielded a highly enriched E16 mAb preparation with a recovery rate comparable to that of Protein A chromatography. The ATPS-enriched E16 retained its structural integrity and was fully functional in binding its target antigen. Notably, HPA-based ATPS was also effective in enriching E16 from plant host proteins when both HPA and E16 were produced in the same leaves, supporting the potential of further streamlining the downstream purification process. Thus, ATPS based on plant-produced HPA in unpurified extract is a cost-effective yet efficient initial capture step for purifying plant-made mAbs, which may significantly impact the approach of mAb purification.

## 1. Introduction

Monoclonal antibodies (mAbs) have dramatically transformed how we approach the treatment of a myriad of diseases [1]. Examples of mAb-therapeutics include cetuximab to treat colorectal cancer [2], avelumab to treat a variety of tumors [3], and the combination of three anti-Ebola mAbs, known as ZMapp, to treat Ebola virus infection [4]. However, the high production cost of mAbs using mammalian cell-based platforms translates into a high market price for approved treatments. Plant-made mAbs have shown great potential in reducing the upstream costs of producing these valuable biologics by eliminating the need for expensive facilities, bioreactors, and culture media required for mammalian cell-based protein production [5]. Although the upstream processing costs can be addressed through plant-based expression platforms [6], the downstream processing remains essentially equivalent to the mammalian cell-produced counterparts [7] and improvements or alternatives to the current methods could help to further reduce the overall production costs.

Although Protein A affinity chromatography is a universally accepted standard purification step in mAb production, the resins for this chromatographic step are expensive and contribute significantly to the overall cost-of-goods of mAb-based drugs [8]. As a result, it is common practice to recycle and reuse the Protein A resin for mAb purification which can reduce column efficiency, as well as result in a reduction in the purity and overall recovery of the antibody [9,10]. The reduction of column efficiency is often attributed to ligand leaching, impurity build-up, restriction of pore access, and blocking access to the Protein A ligand itself [11]. Particularly relevant to plant-made mAbs, studies have shown that the presence of native plant proteins and small-molecule compounds, such as phenolics and alkaloids, in the raw plant extract can impact mAb binding to Protein A in addition to contributing to resin fouling [12,13,14]. Although the presence of these contaminating proteins and compounds can be reduced or eliminated by combining multiple and varying chromatography steps, prolonging the longevity of the Protein A resin of the initial capture step remains a challenge. This calls for the development of alternative strategies that can effectively enrich mAbs from plant extracts at a low cost. 

Aqueous two-phase separation (ATPS) has been a useful method for protein purification by taking advantage of unique characteristics of structurally different polymers to separate biomaterials [15]. In particular, thermo-separating surfactants have been used with hydrophobin tags to purify target proteins from fungi, plants, and insect cells [16,17,18,19]. Recently, it was demonstrated that a novel bifunctional fusion protein could be utilized to capture antibodies in-solution by way of a water-surfactant two-phase system. The bifunctional protein was a genetic fusion of the immunoglobulin-binding domain of Protein A from *Staphylococcus aureus* with a class II hydrophobin, HFBI, from *Trichoderma reesei* [20]. When the bifunctional fusion protein (HPA) was mixed with a mAb in solution, the mAb-HPA complex partitioned to the surfactant-rich phase due to the surfactant-like behavior induced by the hydrophobin moiety of the fusion protein. Upon removal of the residual aqueous phase, where the majority of the production host proteins reside, and after the addition of a low pH buffer to dissociate the mAb from the Protein A binding domain, the mAb was enriched and efficiently separated from most production host proteins [20]. While this strategy was successful in purifying mAbs from media of hybridoma culture, its feasibility in enriching mAbs from systems with more complex mixtures of proteins and other compounds, such as plant extract, has not been demonstrated.

In the current study, we utilized the same bifunctional protein in a water-surfactant two-phase system to serve as an initial capture step in purifying plant-made mAbs. To achieve this, we transiently expressed two protein molecules separately in *Nicotiana benthamiana*: an anti-West Nile virus (WNV) mAb, E16, and the bifunctional fusion protein (HPA) described above, containing the antibody-binding domain of Protein A and the hydrophobin tag. When plant extracts containing E16 and HPA were mixed, E16 was efficiently separated from the majority of plant host proteins by a single ATPS step, yielding a highly enriched mAb preparation with the expected E16 function. Additionally, we explored the feasibility of co-expressing E16 and HPA in the same plant to further streamline the initial capture step. Co-expression of three polypeptides of E16 light chain (LC), heavy chain (HC) and HPA in the same plant produced two properly folded, functional proteins that interact with each other to achieve efficient separation of E16 from the host molecules. ATPS based on plant-produced HPA in unpurified extract has potential to change the way that plant-made mAb purification is approached.

## 2. Results and Discussion

### 2.1. Transient Expression of HPA and E16 mAb in N. benthamiana

**Figure 1:**
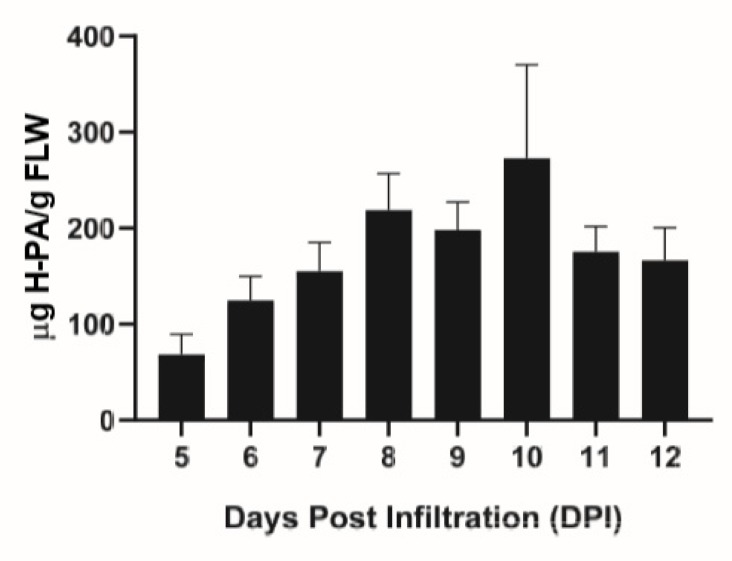
Temporal expression pattern of Hydrogen-Protein A (HPA) fusion protein in *N. benthamiana* plants. Leaves were infiltrated with the HPA construct in the TMV-based MagnICON system and leaf material was collected 5–12 days post infiltration (DPI). Leaf protein extracts were analyzed with an ELISA assay to detect HPA. At least three independent experiments are represented and error bars represent SEM.

Transient expression of the HPA fusion protein in *N. benthamiana* plants was assessed with an enzyme-linked immunosorbent (ELISA) assay. The temporal expression pattern of HPA is similar to that of other proteins using MagnICON vectors [21], increasing up until 8–10 days post infiltration (DPI), where the protein level peaked at 218.7–272.9 μg/g FLW (Figure 1). Previous work with a different protein expression vector reported significantly higher levels of expression of the same protein construct [20]. In addition to using a different expression vector, other factors such as differences in plant growth and agroinfiltration conditions may also contribute to the difference in HPA accumulation in leaves. While optimizing the expression level of this protein in the MagnICON system is required in future work, our current expression system did provide a sufficient amount of HPA fusion protein for analyzing its efficiency in purifying a plant-made mAb. E16 was also transiently expressed in leaves of *N. benthamiana* and its expression levels were quantitated as we reported previously [22]. 

### 2.2. Purification of mAb from Plant Extract via ATPS

**Figure 2:**
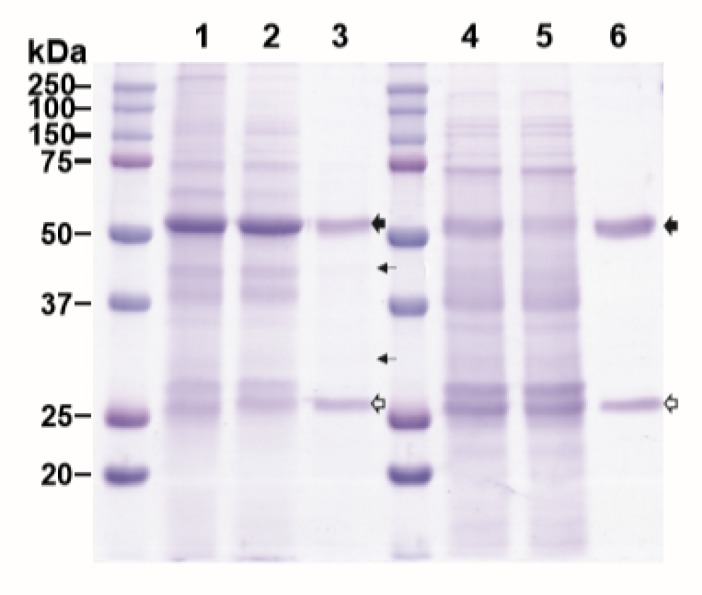
Aqueous two-phase separation (ATPS)-based purification of E16 mAb from *N. benthamiana* leaves. Total soluble proteins were extracted from plants and E16 was purified by ATPS (Lanes 1–3) or Protein A affinity chromatograph (Lanes 4-6), analyzed on a 12% SDS-PAGE gel under reducing conditions, and visualized with Coomassie stain. Lane 1, total leaf soluble proteins; Lane 2, proteins in the upper aqueous phase of ATPS; Lane 3, ATPS-enriched E16; Lane 4, leaf soluble proteins after low pH precipitation; Lane 5, Protein A flow through; Lane 6, Protein A column-purified E16. 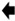: HC, 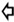: LC, 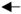: minor impurity, non-mAb bands.

We evaluated the effectiveness of ATPS in purifying plant-made mAb with plant extracts from leaves harvested at 7 DPI. This sampling point was chosen because the levels of expression were high for both HPA (155.2 μg/g FLW) and E16 (339.9 μg/g FLW). A clarified extract from HPA and E16-expressing leaves was mixed volumetrically 1:1 and processed through the ATPS steps. As a reference, an aliquot of the same E16 extract was processed in parallel in Protein A affinity chromatography. As shown in Figure 2, ATPS significantly enriched E16, with HC and LC being the prominent protein bands on the SDS-PAGE. Compared to Protein A chromatography-purified samples, a few more minor non-mAb bands were observed in the lane containing the ATPS-enriched E16 sample (Figure 2, Lane 3 compared to Lane 6). Specifically, densitometry analysis showed that the purity of ATPS-enriched E16 is ~24% less than that of E16 purified by a two-step process of low pH precipitation and Protein A chromatography [22]. Nevertheless, this single ATPS step did remove most of the plant host proteins from the E16 purification feedstream. The recovery rate of E16 through ATPS was 47.7% ± 5.2%, as measured by ELISA, comparable to that of Protein A chromatography [22] but higher than that reported for ATPS purification of mAbs from hybridoma supernatants (24%) [20]. According to our expression data, the molar ratio of HPA:mAb used in ATPS to achieve this recovery rate was approximately 2:1 (1:2 mass ratio). Thus, this favorable ratio [20] may have contributed to the high recovery of E16. Compared with Protein A chromatography, ATPS is simpler and is less expensive. For example, the crude clarified leaf extract in Lane 2 (Figure 2) can be directly used in ATPS for E16 purification. In contrast, the crude extract had to go through a bulk separation step (overnight low-pH precipitation) to remove host proteins (i.e., Rubisco) and other contaminants to yield the starting extract for Protein A chromatography (Figure 2, Lane 4). This bulk separation step is necessary as the removal of most abundant plant host protein (Rubisco) (evidenced by the reduction of the ~ 50 kDa band intensity from Lane 1 to Lane 4) and other impurities are essential to maintain the efficiency of Protein A chromatography [23]. By eliminating the bulk separation step, ATPS saves time and resources, and potentially decreases the loss of mAb due to proteolytic degradation by reducing the time it is exposed to plant host proteolytic enzymes upon homogenization [24]. The ATPS strategy reported here also has advantages over other ATPS methods reported previously [20]. Instead of using purified HPA, the crude plant extract containing HPA was directly mixed with E16-containing extract in our process, eliminating extra steps for HPA purification. This time and cost-saving improvement did not compromise the purity of the resulted mAb, but actually yielded better recovery of mAb (47.7%) from the more complex protein containing plant crude extract than the more cumbersome method (24%) from the hybridoma supernatant [20]. The efficient enrichment and the remarkable rate of recovery supports our ATPS method as a simple, cost effective, yet efficient bulk separation step to enrich plant-made mAbs from most of the host plant proteins. 

### 2.3. Characterization of ATPS-Purified E16 mAb

**Figure 3:**
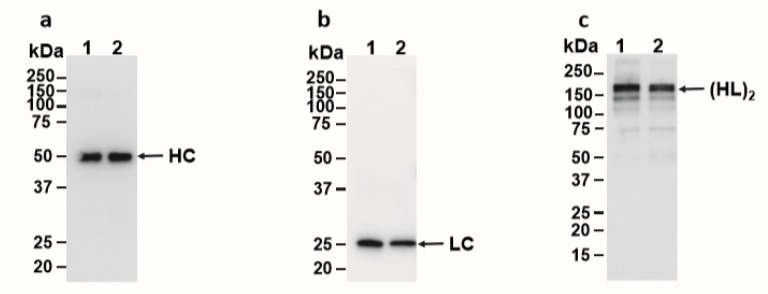
Western blot analysis of ATPS-purified E16. ATPS- or Protein A column-purified E16 was separated on 12% SDS-PAGE gels under reducing (**a** and **b**) conditions or on a 4–20% SDS-PAGE gel under non-reducing (**c**) conditions and blotted onto polyvinylidene fluoride (PVDF) membranes. A goat anti-human gamma chain antibody (**a** and **c**) or a goat anti-human kappa chain antibody (**b**) were incubated with the membranes to detect heavy chain or light chain, respectively. Lane 1, ATPS-enriched E16; Lane 2, Protein A column-purified E16. HC: heavy chain, LC: light chain, (HL)_2_: assembled mAb with two light and heavy chains.

Western blot analysis was used to characterize E16 recovered from ATPS. When the blot was probed with an anti-human gamma-chain antibody, we observed the expected size of HC (50 kDa) similar to that of Protein-A-purified E16 under reducing conditions (Figure 3a), confirming that the 50 kDa band on SDS-PAGE (Figure 2) is indeed the E16 HC and not the Rubisco large subunit. Similarly, the expected 25 kDa LC band was observed under reducing conditions when the blot was incubated with an anti-human kappa-chain antibody (Figure 3b). To ensure that E16 was fully assembled and the integrity was maintained through ATPS, samples were also analyzed under non-reducing conditions. As shown in Figure 3c, both the Protein A column and ATPS-enriched E16 mAbs were observed as a ~179 KDa band. This represents the fully assembled and glycosylated E16 mAb consisting of a heterotetramer of HC and LC as we observed previously [22]. Overall, the enriched mAb is fully assembled and retains its integrity after subjection to ATPS, further supporting our method as an effective capture step for purifying plant-made mAbs. 

### 2.4. Antigen Specificity of ATPS-Purified E16

**Figure 4:**
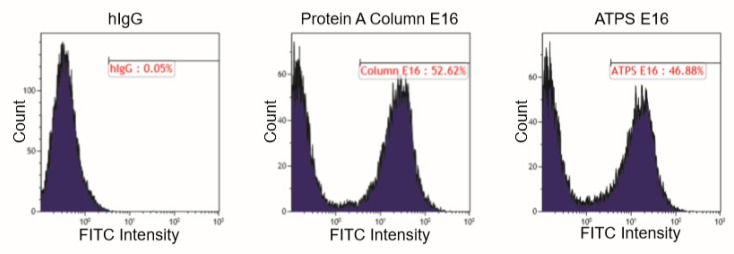
Binding of ATPS-enriched E16 to domain III of WNV E displayed on the surface of yeast cell. Yeast cells displaying domain III of WNV E protein were incubated with ATPS-enriched E16, Protein A column-purified E16 (positive control), or a generic human IgG (hIgG, negative control), and then stained with a Fluorescein isothiocyanate (FITC)-conjugated antibody. Yeast cells were then processed by flow cytometry to monitor the specific binding of E16 to WNV E domain III (DIII).

E16 recognizes a conformational epitope consisting of four discontinuous polypeptides on the surface of the domain III (DIII) of the WNV E protein [25]. Therefore, the binding between E16 and DIII is highly specific and contingent upon the structural integrity of both proteins. To verify that ATPS-enriched E16 retains recognition for its epitope, a binding assay utilizing a yeast display system was performed [22]. Flow cytometric analysis revealed that the incubation of ATPS-purified E16 with WNV DIII-displaying yeast cells resulted in an E16-specific peak that shared a similar percentage of positive yeast cells and fluorescent intensity with that of the Protein A column-purified E16 (Figure 4). This provides additional evidence that ATPS enrichment does not alter the mAb’s specificity to its antigen, further supporting the use of this method for purifying mAbs that require functional integrity.

### 2.5. ATPS with Co-expression of E16 and HPA in Plants

**Figure 5:**
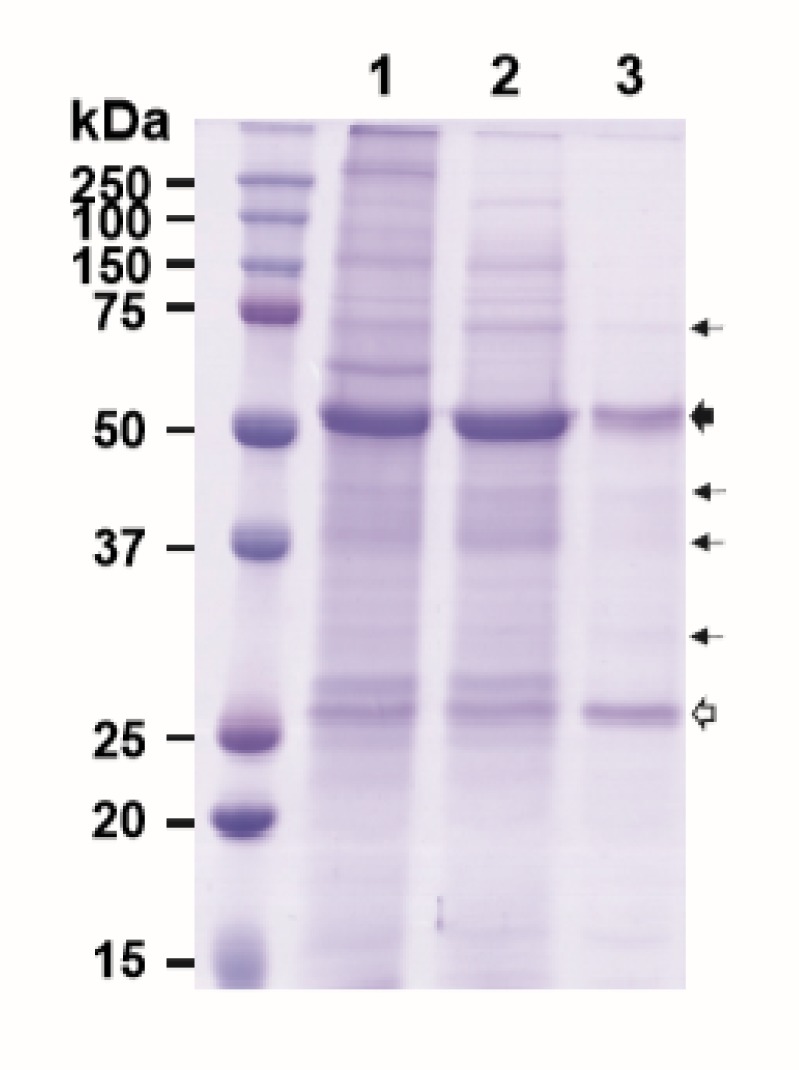
ATPS purification of E16 with HPA co-expressed in the same *N. benthamiana* leaf. Total leaf soluble proteins were extracted from leaves co-expressing both E16 and HPA. The plant extract was then processed through ATPS to enrich E16. ATPS fractions were analyzed on a 12% SDS-PAGE gel under reducing conditions and visualized with Coomassie stain. Lane 1, total leaf soluble proteins; Lane 2, proteins in the upper aqueous phase of ATPS; Lane 3, ATPS-enriched E16. 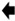: HC, 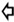: LC, 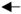: minor impurity, non-mAb bands.

Purification of plant-made mAbs could be further streamlined by expressing both the mAb and its purifying reagent molecule, such as HPA, in the same plant. Thus, we investigated the feasibility of co-expressing E16 and HPA together in the same plant and using the co-expressed proteins to purify E16 via ATPS. As the production of incompletely assembled antibodies would be deleterious to the immune response, the in vivo assembly of the antibody is a tightly controlled process involving the sequential interaction between the HC, LC, and associated chaperons [26]. Specifically, the early formation of HC dimers is crucial for the subsequent binding of LCs and any interference of this event may affect the overall assembly of the full antibody [26,27]. Our previous results have shown that the accumulation levels of a mAb in plants is greatly reduced when expressed in the format of a recombinant immune complex (RIC) compared to its parent IgG [28,29]. These results support the hypothesis that the *in planta* interaction of mAb HC-antigen fusion and its antigen binding domain (Fab) between RICs interferes with HC assembly. To avoid the potential interaction of E16 HC and HPA *in planta* which may also interfere with E16 HC and LC assembly, we took advantage of the phenomenon of “competing replicons” of the MagnICON vectors [30,31,32]. By cloning the E16 HC and HPA genes into a vector that is based on the same TMV backbone, only the HC or HPA, but not both would be preferentially expressed in any given cell to avoid their interaction. We anticipated that the LC, being delivered in a non-competing PVX-based vector, would be expressed in all infiltrated cells. Only in the cells that preferentially expressed the HC over HPA would a fully assembled E16 be produced. Upon homogenization of the leaf material, the fully assembled E16 would bind to HPA, while any unassembled LC would not associate with any HPA, thus facilitating an initial capture and enrichment of assembled E16 via ATPS from plant crude extract. Indeed, our results indicated that we were able to enrich E16 that was co-expressed with HPA in *N. benthamiana* leaves, albeit with a lower purity (9.4% less pure by densitometry analysis) than when they were expressed in separate plants (Figure 5, Lane 3). The yield of E16 is lower, as only a percentage of infiltrated cells would express E16 HC due to the competition from HPA. The low yield of E16 may also have altered the ratio of E16 to HPA, contributing to lower ATPS efficiency and lower E16 purity. Therefore, further optimization to better balance the expression of each molecule *in planta* is needed to improve the yield and purity of mAbs by this approach. Nevertheless, to our knowledge, this is the first demonstration of co-expression of a mAb and a bifunctional protein capable of binding the mAb in the same plant, and furthermore, the successful partitioning and enrichment of the mAb by a capturing molecule in a simple two-phase extraction system. This provides evidence for potentially further enhancing the efficiency of the purification scheme proposed in this study.

Scalability is one of the deciding factors for the adaptation of ATPS in the commercial setting for mAb production. The scalability of ATPS has been demonstrated in processing various protein products with high purity and recovery [33]. Moreover, it has been shown that ATPS can be integrated seamlessly with other downstream processing techniques [34]. In a recent study, the scalability of HPA-based ATPS for mAb production has been demonstrated [20]. This further suggests the potential application of using HPA-containing plant-extract in purifying plant-made mAbs.

## 3. Materials and Methods

### 3.1. Materials

The salts for phosphate buffered saline, sodium L-ascorbate, carbonate, bicarbonate, citric acid, yeast nitrogen base without amino acids, yeast synthetic drop-out medium supplements, ammonium persulfate, tetramethylethlyenediamine (TEMED), glucose and galactose were all purchased from Sigma-Aldrich (St. Louis, MO, USA). Coomassie Brilliant Blue R-250 and OmniPur® phenylmethylsulfonyl fluoride (PMSF) were purchased from EMD Chemicals (Gibbstown, NJ, USA). Ethylenediaminetetraacetic acid (EDTA), Tween-20, Tris-base and sodium dodecyl sulfate (SDS) were purchased from Fisher Scientific (Fair Lawn, NJ, USA). All detection antibodies used for ELISAs and Western blots were purchased from SouthernBiotech (Homewood, AL, USA). Plates used for ELISAs were purchased from Corning (Kennebunk, ME, USA). The KPL 3, 3′, 5, 5′–tetramethylbenzidine (TMB) peroxidase substrate kit for ELISA was purchased from SeraCare (Milford, MA, USA). Proteomics grade Triton X-114 was purchased from Amresco® Chemicals (Solon, OH, USA). Electrophoresis grade glycine was purchased from MP Biomedicals LLC (Solon, OH, USA). 2-methylpropan-1-ol (isobutanol) was purchased from Acros Organics (Fair Lawn, NJ, USA). Centrifugal filter units were purchased from Millipore (Billerica, MA, USA). Pierce detergent removal spin columns, Millipore Express PLUS 0.22μm vacuum membranes and Pierce ECL Western blotting substrate were all purchased from Thermo Scientific (Rockford, IL, USA). MabSelect resin was purchased from GE Healthcare (Upsala, Sweden). 30% (29:1) acrylamide/bis solution and polyvinylidene fluoride (PVDF) membrane were purchased from Bio-Rad (Hercules, CA, USA).

### 3.2. Bacterial Strains and Growth

The coding sequence of HPA [20] was cloned into the MagnIcon vector pICH11599 and transformed into *Agrobacterium tumefaciens* GV3101 cells as described previously [35].

### 3.3. Agroinfiltration

*N. benthamiana* plants were grown and infiltrated with target gene-harboring *A. tumefaciens* GV3101 strains as previously described [36]. Specifically, *A. tumefaciens* strains containing the E16 HC 3′ module, E16 LC 3′ module [22], their corresponding 5′ modules, and the integrase construct [37] in the MagnICON system were co-infiltrated into leaves of 5-7-week old *N. benthamiana* with a combined OD_600_ of 0.74. GV3101 strains containing the HPA 3′ module, the 5′ module, and the integrase module were co-infiltrated into leaves with a final OD_600_ = 0.9.

For co-expression of E16 and HPA in the same leaves, the six strains of GV3101 required for E16 HC, LC and HPA expression were co-infiltrated into the leaves *N. benthamiana* plants with a final OD_600_ of 0.98 as described previously [38].

### 3.4. HPA ELISA

HPA-expressing leaves were homogenized in 1× PBS (8 mM Na_2_HPO_4_, 2 mM KH_2_PO_4_, 15 mM NaCl, 2.5 mM KCl) pH 7.4, supplemented with 10 mg/mL of sodium L-ascorbate, 2 mM PMSF, and 1mM EDTA in a fresh leaf mass to buffer volume ratio of 1:1.5 [39]. The plant extract was then clarified by centrifugation twice at 16,873× *g* for 10 min at 4 °C. Samples were then diluted in 100 mM bicarbonate buffer (pH 9.2) along with the HPA standard, ranging from 50 ng/mL to 0.78 ng/mL in a 96-well plate. The plate was then incubated at 37 °C for 2 h. After blocking with 5% skim milk in PBST (1× PBS with 0.05% Tween-20), generic human IgG was added to the plate to interact with the antibody-binding domain of HPA coated on the plate. The plate was incubated at 37 °C for 1 h, and a horseradish peroxidase-conjugated goat anti-human kappa chain was added as the detection antibody. The plate was developed with TMB and then read at 450nm as previously described [40]. Protein expression level was then calculated as micrograms of recombinant protein per gram of fresh leaf weight (FLW).

### 3.5. Aqueous Two-Phase Separation

Leaves expressing each recombinant protein (HPA or E16) were harvested at 7 days post infiltration (DPI) and homogenized in extraction buffer separately in a blender as described [41]. After clarification by centrifugation, equal volumes of E16 and HPA infiltrated leaf extract were mixed and incubated at 4 °C for 1 h. The mixed extract was then diluted 1:10 in PBS pH 7.4 and incubated in a 26 °C water bath for 5 min. Triton X-114 was added to 1mL of the pre-warmed mixed extract at a concentration of 4% *w*/*v* and mixed well. Phase separation was achieved by a 5-min incubation in at 26 °C. The upper aqueous phase was removed and 0.5 mL of 100 mM glycine-HCl (pH 2.0) was added to the lower detergent-rich phase and thoroughly mixed to dissociate E16 from HPA. After phase separation as described above, the upper phase containing the enriched E16 was transferred to another tube and pH adjusted to pH 7.0 with 1M Tris-base. The HPA fusion protein was recovered by adding isobutanol to the lower detergent-rich phase as described previously [20]. The same ATPS procedure was followed when HPA and E16 were co-expressed in the same leaf, except there was no separate homogenization and mixing of leaf extracts. When necessary, the enriched E16 fractions were concentrated with a 100K MWCO centrifugal filter unit and passed through a detergent removal spin column before further analysis.

### 3.6. Protein A Affinity Chromatography of E16

For use as a reference standard, plant-expressed E16 was also purified by Protein A affinity chromatography as described previously [42]. Briefly, control leaves infiltrated with the E16 construct were homogenized as described above, with the exception that the extraction buffer pH was 5.2. After clarification by centrifugation, the plant extract was re-adjusted to pH 5.2 and further incubated overnight at 4 °C. After removal of the precipitated plant host proteins by centrifugation, the extract was adjusted to pH 7.2, filtered through a Millipore Express PLUS 0.22μm membrane. The filtered extract was then loaded onto a Protein A column with MabSelect resin at a rate of 0.8-1.2mL/minute. E16 was eluted from the Protein A column with 100mM citric acid, which was then neutralized with 1M Tris-base.

### 3.7. SDS-PAGE and Western Blotting

Fractions of the starting extract and the flow through (Protein A column) or upper phase (ATPS), along with equivalent amounts of purified E16 from both methods were subjected to reducing SDS-PAGE on a 12% gel and stained with Coomassie Brilliant Blue R-250. For co-expression, only the ATPS samples were included. The purity of E16 mAb was determined by imaging and quantitating Coomassie blue-stained protein bands on SDSPAGE using a densitometer as described previously [43]. For Western blot analysis, ATPS and column-purified E16 samples were separated via SDS-PAGE on 12% or 4–20% gradient gels under reducing or non-reducing conditions, respectively, and then transferred to a PVDF membrane. After blocking with 5% milk in PBST, the membranes were probed with either goat anti-human gamma chain-HRP or kappa chain-HRP antibody diluted in 1% milk in PBST. After washing with PBST, Pierce ECL Western blotting substrate was mixed according to the manufacturer’s instructions and used to cover the membrane. The ImageQuant LAS 4000 imager and associated software was then used to image the blots. 

### 3.8. E16 ELISA for ATPS E16 Recovery

Recovery of E16 purified from ATPS was quantified by an ELISA as previously described [42]. Briefly, 96-well plates were coated with the WNV domain III (DIII) of the E protein [41]. After blocking with 5% milk in PBST, fractions from the various ATPS purification steps were added to the wells, along with mammalian cell-produced E16 as a standard [37]. A goat anti-human kappa chain-HRP was used as the detection antibody. The plate was then developed with TMB and read with a plate reader at 450nm. E16 recovery by ATPS was calculated as the percentage of purified E16 obtained from the total E16 in the mixed plant extract.

### 3.9. Yeast Display Assay

The yeast display assay was adapted from our published procedure [22]. *Saccharomyces cerevisiae* EBY100 cells harboring the gene for WNV DIII in the yeast surface display vector, pYD1, were grown in synthetic yeast medium with 2% glucose at 30 °C until log phase growth was reached (OD_600_ 2-5). Cells were then grown an additional 36 h at room temperature in synthetic yeast medium with 2% galactose to induce expression and display of DIII on the yeast surface. Cells were collected, washed, and incubated with ATPS-enriched E16, Protein-A-purified E16 (positive control) or a generic human IgG (negative control). Cells were then stained with goat anti-human IgG-FITC and counted with a Gallios flow cytometer.

## 4. Conclusions

The use of plants as a production platform for mAb therapeutics has great potential to not only reduce the upstream cost of production [6], but also provide opportunities to improve efficacy and safety [40,44,45]. However, the downstream processing costs between plant and cell culture-based platforms remain essentially equivalent, highlighting the need for alternatives or improvements to the current methods for mAb purification [46]. Removing as many contaminating molecules as possible by introducing an efficient yet inexpensive initial capture step, as described here, will prolong the longevity of the Protein A resins in the affinity chromatography step that are the current gold standard for mAb purification. In this study, we built upon the novel concept of utilizing a bifunctional fusion protein to capture a mAb in an ATPS to reduce downstream costs of purifying mAbs from plants. By expressing both the E16 mAb and the HPA fusion protein in plants, directly mixing plant extracts in a controlled ratio, and enriching the mAb through the facile ATPS method described here, we were able to produce a highly enriched E16 mAb with a recovery rate comparable to that obtained from the conventional Protein A affinity chromatography. Furthermore, the recovered mAb was fully assembled and fully functional by retaining recognition of its target antigen. This highly enriched E16 preparation not only can be easily purified to homogeneity by the Protein A affinity chromatography step but may also increase the longevity of the Protein A resin due to the reduction of host proteins in the feedstream of the affinity chromatography. Of note is the demonstration of a mAb and the HPA fusion protein being co-expressed within the same plant and the subsequent enrichment of the mAb via ATPS with an inexpensive detergent. These steps towards improving the purification methods of plant-made mAbs are promising and warrant further optimization and studies on a larger scale to validate the utility of this system.
